# Adiponectin C1q/Tumor Necrosis Factor-Related Protein 13 (CTRP13) Protects against Renal Inflammation and Fibrosis in Obstructive Nephropathy

**DOI:** 10.3390/biomedicines12010051

**Published:** 2023-12-25

**Authors:** Yongxia Li, Wenzhe Wang, Changxuan Liu, Min Zeng, Li Xu, Rong Du, Cheng Wang

**Affiliations:** 1 Department of Rheumatology, Union Hospital, Tongji Medical College, Huazhong University of Science and Technology, Wuhan 430022, China; liyongxia@zxhospital.com; 2 Department of Nephrology, The Central Hospital of Wuhan, Huazhong University of Science and Technology, Wuhan 430022, Chinaliuliu820723@163.com (C.L.);

**Keywords:** kidney, fibrosis, inflammation, CTRP13

## Abstract

Renal inflammation and fibrosis are the important pathological phenomena associated with obstructive nephropathy. However, the underlying mechanism associated with this disease has yet to be fully elucidated. The present study, therefore, aimed to investigate the effects mediated by C1q/tumor necrosis factor-related protein 13 (CTRP13) on renal inflammation and fibrosis in addition to elucidating the underlying mechanism. To meet this aim, a mouse unilateral ureteral obstruction (UUO)-mediated renal dysfunction model was established. In addition, hematoxylin–eosin staining (H&E) staining and immunofluorescence experiments as well as Western blotting and reverse transcription quantitative (RT q) PCR analyses were performed. Recombinant CTRP13 was used to investigate the role of CTRP13 in chronic renal inflammation and fibrosis. A decreased expression level of CTRP13 was identified in the plasma of patients with renal fibrosis and in UUO-model mice. The renal histopathological and functional analyses revealed that CTRP13 could both reverse UUO mediated renal dysfunction and ameliorate the conditions of tubulointerstitial fibrosis and tubular injury. Additionally, CTRP13 was found to inhibit the expression levels of extracellular matrix proteins and proinflammatory mediators. In terms of the underlying mechanism, the protective effects on inflammation and fibrosis of the kidneys of CTRP13-treated mice undergoing UUO were found to be associated with the inactivation of the TGF β/Smad and NF κB p65 signaling pathways. Taken together, these findings have suggested that CTRP13 fulfills a vital role in the progression of obstructive nephropathy, thereby uncovering brand new insights into possible leads for the therapeutic treatment of chronic kidney disease (CKD).

## 1. Introduction

Chronic renal fibrosis, which leads to end-stage renal failure, is a major worldwide health problem that results in high mortality and morbidity rates, and its incidence has increased globally on an annual basis [1,2,3,4]. The renal fibrosis process is dynamic, characterized by an increase in interstitial proinflammatory cell infiltration, the activation of renal interstitial fibroblasts, and the deposition of extracellular matrix (ECM) proteins, eventually leading to the loss of renal function and damage to renal parenchyma [3,5]. However, at present, effective treatments designed to prevent the progression of kidney fibrosis and to delay chronic-kidney-disease-associated complications remain limited. Consequently, an improvement in our understanding of the mechanism(s) associated with renal fibrosis and identifying relevant targets for the prevention of renal fibrosis’ progression, are both necessary in order to provide the theoretical basis underlying the treatment of chronic kidney disease (CKD).

C1q/tumor necrosis factor-related proteins (CTRPs), which share several structural characteristics in common, are conserved members of the adiponectin family. The CTRP family serves multiple roles in regulating the pathophysiology of metabolism in addition to the immune and vascular systems [6,7]. To date, among the 15 identified CTRPs, it has been reported that the sequence of CTRP13 only differs in one amino acid comparing the human and mouse forms [8]. As an adipokine, CTRP13 has also been shown to mediate glucose metabolism in hepatocytes, myotubes, and adipocytes [8,9]. A study published recently by our group [10] demonstrated that the supplementation of CTRP13 inhibited vascular calcium accumulation in end-stage renal failure. However, a precise knowledge both of the association between chronic kidney disease and CTRP13 as well as the underlying mechanism(s) remains lacking. The aims of the present study were therefore to investigate whether there is an association between CTRP13 and unilateral ureteral obstruction (UUO)-mediated renal fibrosis and tubulointerstitial inflammation and to elucidate the potential underlying mechanism(s).

In our recent work, dynamic changes in CTRP13 were observed during the development of kidney fibrosis, and, subsequently, the protective effects of CTRP13 on kidney fibrosis together with the underlying mechanism were explored through ectopic CTRP13 infusion. The results obtained suggested that CTRP13 is a critical regulator that serves as a ‘brake’ to minimize kidney dysfunction and fibrosis in the renal injury process.

## 2. Materials and Methods

### 2.1. Establishment of the UUO-Induced CKD Model

C57BL/6 mice were purchased from the Model Animal Research Center of Huazhong University of Science and Technology. The mice were provided with free access to diet and water and housed under conditions of 22 °C temperature, 50–60% humidity, and regular lighting conditions (12 h light/dark cycles). The experimental C57BL/6 mice (male mice aged 8–10 weeks) were subjected to double-ligation of the left ureter. Renal fibrosis was induced following a UUO operation, as described previously [11]. In brief, mice were anesthetized by the intraperitoneal injection of ketamine (100 mg/kg) and xylazine (10 mg/kg) and were placed on a temperature-controlled operating table to maintain their body temperature at 37 °C. Through a midline incision in the abdomen, the right proximal ureter was exposed and ligated at two separated points using 3-0 black silk. The sham operation was performed using the same method but without the ligation of the ureter. Following surgery, the mice were subsequently randomly assigned to two groups: (i) vehicle injection and (ii) CTRP13 injection. Vehicle (normal saline) or recombinant human CTRP13 protein (Aviscera Bioscience, Inc., Santa Clara, CA, USA; 200 mg/kg) was intraperitoneally injected into the mice every other day for 2 weeks. After the completion of the experiment, the mouse kidneys were obtained through an abdominal incision under anesthesia, and, subsequently, the mice were euthanized (the collected blood and kidneys were stored at −80 °C prior to further analysis, and all mice were anesthetized using 2%, and sacrificed with 5% isoflurane). Death was confirmed when the mice had stopped breathing and after they had lost all of their bodily responses upon receiving a stimulus. All animal experimental protocols were approved by the Ethics Committee of Huazhong University of Science and Technology (Institutional Animal Care and Use Committee (IACUC) no. #2948).

### 2.2. Histology Staining

With the scheduled necropsy, the left kidney was removed, fixed in cold 70% ethanol, and routinely embedded in paraffin. The blocks were cut into 5 μm sections and subsequently stained using H&E and Masson’s trichrome staining. Image Pro-Plus 6.0 software was employed to analyze the quantification of the fibrotic area.

The mouse kidney sections were incubated with anti-α-smooth muscle actin (anti-α-SMA) (1:200 dilution; cat. no. 50,513), anti-vimentin (1:200, cat. no. 5741), anti-collagen I (1:200, cat. no. 81,375), anti-F4/80 (1:200, cat. no. 30,325), or anti-phospho-NF-κB p65 (1:200, cat. no. 3033) antibody overnight at 4℃ (all the antibodies were purchased from Cell Signaling Technology, Inc., Danvers, MA, USA). The slides were subsequently stained using a secondary antibody for 1 h at 37 °C and visualized on a ZEISS confocal microscope (Zeiss AG, Oberkochen, Germany).

Renal injury, including tubular and glomerular deformation and tubular epithelial cell detachment, was assessed using a semi-quantitative analysis method, as described previously [12], according to the following scoring system: 0, no abnormality; 1, renal impairment < 10%; 2, renal impairment < 25%; 3, renal impairment < 50%; 4, renal impairment < 75%; and 5, renal impairment > 75%. Semi-quantitative analysis of the fibrotic area was assessed by calculating the percentage of blue staining over the entire view [11]. Both semi-quantitative assessments were performed on 10 randomly selected non-overlapping areas under an optical microscope. These assessments were assessed in a blind manner by the researchers.

### 2.3. Biochemical Detection

Urine and serum samples were retained for assaying the levels of blood urea nitrogen (BUN), 24-hour urine protein (24-h UP) and serum creatinine (SCr) using the corresponding kits [13] (Nanjing Jiancheng, Nanjing, China; cat. nos. C035-2-1, C013-2-1, and C011-2-1, respectively).

### 2.4. Cell Culture and Treatment

NRK-49F cells (i.e., renal interstitial fibroblasts) were obtained from Jennio Biotech Co., Ltd. (Guangzhou, China). The cells were cultured in DMEM supplemented with 10% fetal bovine serum and were incubated in the presence of 5% CO_2_ at 37 °C [14]. For those cells that were to be treated with TGF-β1, 5 ng/mL TGF-β1 (cat. no. 240-B-002; R&D Systems, Inc., Minneapolis, MI, USA) was added to the NRK-49F cells at the indicated times on the next day after the cells had reached 40% confluence.

### 2.5. Western Blot Analysis

Total protein was extracted from the kidney and cultured cells using RIPA lysis buffer. Western blot analysis was performed essentially as described previously [15,16]. Total protein from mouse tissues were resolved by SDS-PAGE. Antibodies against monocyte chemoattractant protein-1 (MCP-1) (1:1000; cat. nos. 39,091 and 41,987; Cell Signaling Technology, Inc., Danvers, MA, USA), interleukin (IL)-1β (1:500; cat. no. 12,242; Cell Signaling Technology, Inc., Danvers, MA, USA), IL-6 (1:1000; cat. nos. 12,912 and 12,153; Cell Signaling Technology, Inc., Danvers, MA, USA), vimentin (1:1000; cat. no. 5741l; Cell Signaling Technology, Inc., Danvers, MA, USA), α-SMA (1:1000; cat. no. 50,513; Cell Signaling Technology, Inc., Danvers, MA, USA), collagen I (1:200; cat. no. 81375; Cell Signaling Technology, Inc., Danvers, MA, USA), TGF-β (1:1000; cat. no. 3711; Cell Signaling Technology, Inc., Danvers, MA, USA), Smad7 (1:1000; cat. no. 9523; Cell Signaling Technology, Inc., Danvers, MA, USA), Smad7 (1:1000; cat. no. 516288; Zen-Bioscience, Durham, NC, USA), Smad3 (1:200; cat. no. 9523; Cell Signaling Technology, Inc., Danvers, MA, USA), NF-κB p65 (1:1000; cat. no. 8242; Cell Signaling Technology, Inc., Danvers, MA, USA), phospho-Smad3 (1:1000; cat. no. 9520; Cell Signaling Technology, Inc., Danvers, MA, USA), NF-κB phosphorylated-p65 (1:1000; cat. no. ab76302; Abcam, Cambridge, UK), and β-actin (1:2000; cat. no. ab8226; Abcam, Cambridge, UK) were used as the primary antibodies. Subsequently, the membranes were incubated with blocking serum and horseradish peroxidase (HRP)-labeled secondary antibody. Finally, immunoreactivity was visualized using enhanced chemiluminescence reagents (Bio-Rad Laboratories, Inc., Hercules, CA, USA), and a semiquantitative analysis was conducted using Fiji ImageJ software.

### 2.6. Reverse Transcription Quantitative (RT-q) PCR Analysis

Total RNA was isolated from cell samples using an Invitrogen™ TRIzol^®^ reagent kit (Thermo Fisher Scientific, Inc., Waltham, MA, USA), following the manufacturer’s protocol. RNA was reverse-transcribed into cDNA using a PrimeScript™ RT Reagent Kit (Takara Bio, Inc., Kusatsu, Japan) followed by qPCR analysis using SYBR™ Green PCR Master mix (Promega Corporation, Madison, WI, USA) on a CFX96 PCR Detection System. The sequences of the primers used in the present study were as follows: E-cadherin, forward, 5′-GGCCCAGGAGCTGACAAAC, and reverse, 5′-CC AGAGGCTGCGTCACTTTC; vimentin, forward, 5′-ATGAAAGTGTGGCTGCC AAGAAC, and reverse, 5′-GTG ACTGCACCTGTCTCCGGTA; and α-SMA, forward, 5′-CCTCTTCCAGCCATCTTTCAT, and reverse, 5′-CGAGAGGACGTTGTTAGCAT AG. Quantification of the cDNA was determined using the 2^−ΔΔCq^ method. 

### 2.7. Statistical Analysis

Data are presented as the mean ± SD for at least 3 independent experiments. The significance was calculated via Student’s t test (for comparisons of 2 groups) or one-way ANOVA with Bonferroni post hoc test (for comparisons of ≥3 groups). All statistical analyses were performed using GraphPad Prism v.6.0. *p* < 0.05 was considered to indicate a statistically significant difference.

## 3. Results

### 3.1. Plasma CTRP13 Levels Are Lower during the Progression of Renal Fibrosis

**Figure 1:**
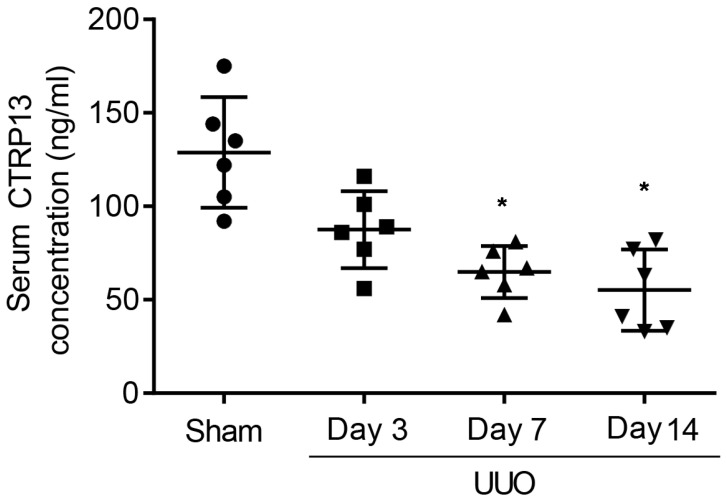
Plasma CTRP13 levels were decreased in the UUO mice with renal fibrosis. The concentration of CTRP13 in the mouse kidney at 3, 7, and 14 days after UUO surgery. * *p* < 0.05 versus Sham group.

To investigate whether CTRP13 was associated with the pathogenesis of CKD, first, changes in the serum level of CTRP13 during renal fibrosis’ development in UUO-model mice were investigated. These experiments revealed that the plasma CTRP13 levels progressively decreased following UUO injury, reaching a minimal point at 14 days (Figure 1 and Table S1). These results indicated that, during the development of kidney fibrosis, the level of CTRP13 gradually decreased, suggesting that CTRP13 may be involved in renal fibrosis.

### 3.2. CTRP13 Treatment Leads to an Improvement in UUO-Mediated Renal Dysfunction

**Figure 2:**
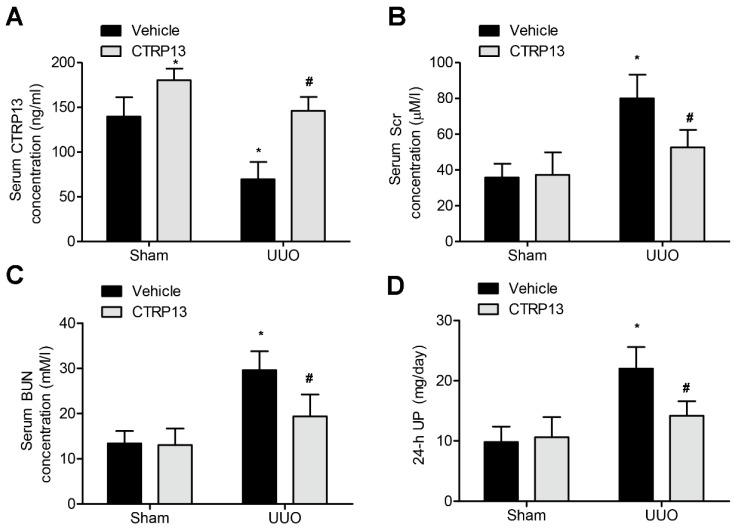
CTRP13 improves UUO-mediated renal dysfunction.C57BL/6 mice were subjected to UUO-induced experimental renal fibrosis model and then were injected with or without recombinant CTRP13 every other day for 2 weeks. (**A**) Plasma CTRP13 levels in the mice. (**B**–**D**) CTRP13-reduced BUN, 24 h UP, and SCr in the UUO mouse model. * *p* < 0.05 versus Sham+Vehicle group. # *p* < 0.05 versus UUO+Vehicle group.

Subsequently, the effect of administering CTRP13 on the UUO-mediated renal fibrosis model in C57BL/6 mice was investigated. CTRP13 was injected into mice every other day for 2 weeks, and the serum CTRP13 levels were found to have been successfully increased (Figure 2A and Table S2). As shown in Figure 2B,D and Table S2, after 2 weeks of treatment, the levels of BUN, SCr, and 24 h UP were statistically markedly different comparing between the sham and the UUO groups. Furthermore, CTRP13 treatment led to a reduction in the levels of Scr and Bun as well as those of 24 h UP, suggesting that ectopic CTRP13 infusion led to the alleviation of UUO-mediated renal function deterioration in vivo.

### 3.3. CTRP13 Ameliorates Kidney Injury and Fibrosis in UUO-Model Mice

**Figure 3:**
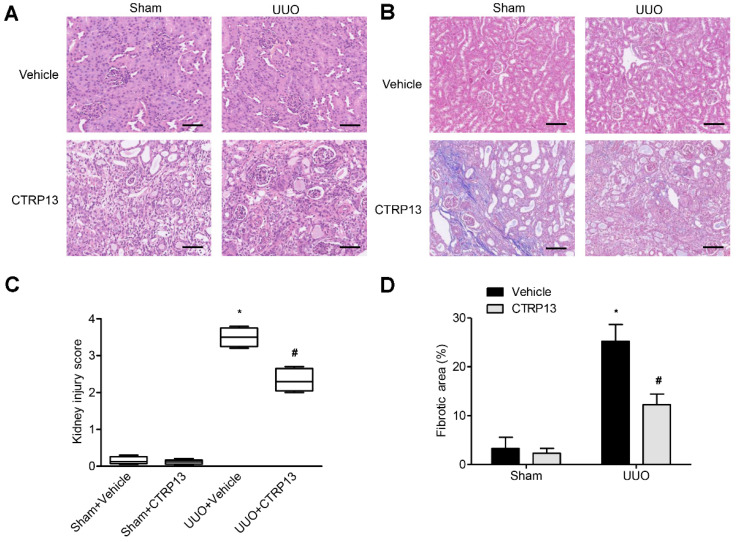
CTRP13 protects UUO-mediated renal injury. (**A**,**B**) Representative Masson and HE staining of kidney tissue sections. After UUO surgery, the tubules were markedly dilated, with extensive interstitial fibrosis and massive inflammatory cell infiltration. After CTRP13 intervention, histopathological damage and interstitial fibrosis were reduced, and glomerular and tubular malformations were reversed. Scale bar = 50 μm. (**C**,**D**) Results of semiquantitative analysis of fibrotic area and renal injury scores. * *p* < 0.05 versus Sham+Vehicle group. # *p* < 0.05 versus UUO+Vehicle group.

Kidney fibrosis is identified by tubule atrophy, interstitial chronic inflammation and fibrogenesis, glomerulosclerosis, and vascular rarefaction [17]. As shown in Figure 3A,B, kidney fibrosis and injuries were assessed via Masson and H&E staining. Following UUO surgery, the tubules were found to be markedly dilated, with extensive interstitial fibrosis and massive inflammatory cell infiltration. Following intervention with CTRP13, histopathological damage and interstitial fibrosis were found to be reduced; moreover, glomerular and tubular malformations were reversed. The injury scores of the kidneys and the area of fibrosis both indicated that CTRP13 exerts a protective effect on UUO-mediated kidney fibrosis and pathological injury (Figure 3C,D).

### 3.4. CTRP13 Inhibits UUO-Induced ECM Accumulation

**Figure 4:**
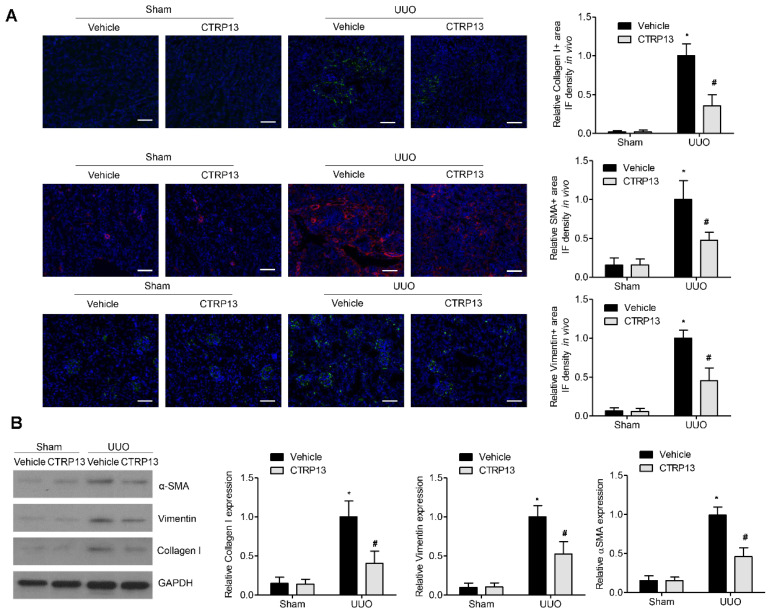
CTRP13 inhibits ECM accumulation in UUO mice. (**A**) Representative immunofluorescence staining of VIM, COL I, and α-SMA in the kidney of UUO mice. Scale bar = 50 μm. (**B**) Representative Western blot analysis of VIM, COL I, and α-SMA in the kidney of UUO mice. * *p* < 0.05 versus Sham+Vehicle group. # *p* < 0.05 versus UUO+Vehicle group.

Fibrosis occurs when wound healing is deregulated, leading to excessive ECM protein accumulation. Accumulation of the ‘classical’ ECM proteins, such as collagen I, vimentin, and α-SMA, is the central feature during fibrogenesis [18]. As shown in Figure 4B, in contrast with the sham group, the levels of collagen I, vimentin, and α-SMA were significantly upregulated following UUO surgery, whereas the administration of CTRP13 prevented the UUO-mediated upregulation of collagen I, vimentin, and α-SMA. These findings were corroborated by the immunofluorescence assay experiments, wherein significant decreases in the expression levels of the ECM proteins at 14 days following surgery were identified in the kidneys of the CTRP13-treated UUO-model mice (Figure 4A). Taken together, these findings suggested that CTRP13 can effectively protect against fibrosis by reducing the accumulation of ECM in the UUO-model mice.

### 3.5. CTRP13 Regulates the TGF-β1/Smad Signaling Pathway in UUO-Model Mice

**Figure 5:**
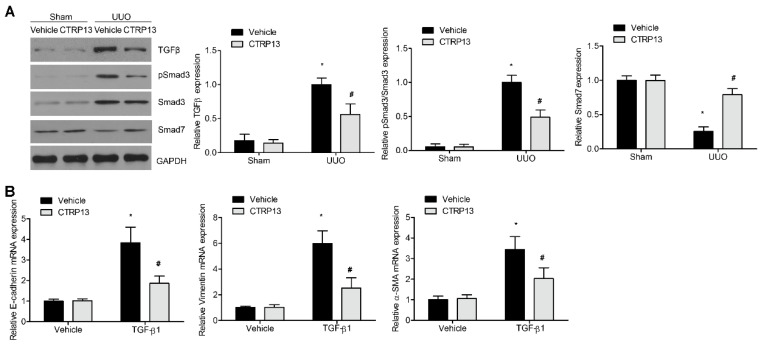
CTRP13 mediates UUO-induced TGF-β1/smads signaling pathway transduction. (**A**) Representative Western blot analysis of TGF-β1, Smad3, phosphorylated Smad3, and Smad7 in the kidney of UUO mice. (**B**) Quantitative real-time PCR analysis of α-SMA, VIM, and COL I in NRK-49F cells after TGF-β1 treatment. * *p* < 0.05 versus Sham+Vehicle or Vehicle group. # *p* < 0.05 versus UUO+Vehicle or TGF-β1+Vehicle group.

To explore the potential protective effects of CTRP13 on kidney fibrosis in the UUO-model mice, the role of the TGF-β1/Smad pathway was analyzed. Compared with the sham group, the UUO-model kidneys showed a marked increase in the levels of TGF-β1 and Smad3 phosphorylation. Interestingly, supplementation of CTRP13 led to an attenuation of the increase in phosphorylated Smad3 and TGF-β1 in the UUO-model mice. Smad7, another downstream negative modulator of TGF-β1, was shown to be downregulated during renal fibrosis (Figure 5A). CTRP13 treatment was found to reverse the UUO-regulated downregulation of Smad7 (Figure 5A). Subsequently, whether CTRP13 could modulate renal matrix protein production and myofibroblast activation in normal rat fibroblasts was investigated. After 24 h of incubation, TGF-β1 treatment led to a marked increase in the expression levels of collagen I, vimentin, and α-SMA, whereas treatment with CTRP13 significantly decreased the TGF-β1-induced upregulation of collagen I, vimentin, and α-SMA (Figure 5B). Taken together, these data suggested that CTRP13 exerts a major role on the TGF-β1/Smad pathway, which could be an important mechanism underpinning the protective function of CTRP13 on the progression of kidney ECM deposition and fibrosis.

### 3.6. CTRP13 Suppresses Renal Inflammation in UUO-Model Mice

**Figure 6:**
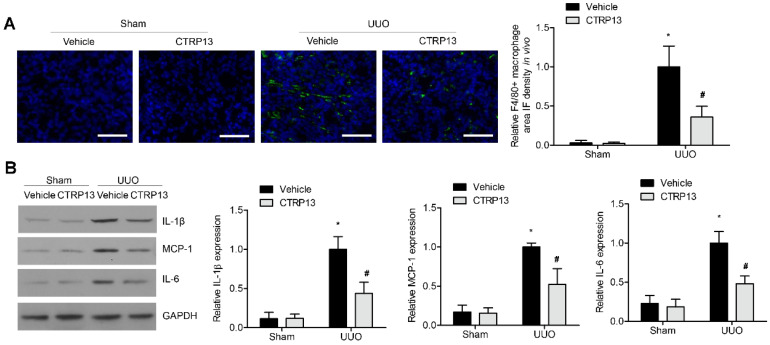
CTRP13 inhibits renal inflammation in UUO mice. (**A**) Representative immunofluorescence staining of F4/80 in the kidney of UUO mice. Scale bar = 50 μm. (**B**) Representative Western blot analysis of MCP-1, IL-1β, and IL-6 in the kidney of UUO mice. * *p* < 0.05 versus Sham+Vehicle group. # *p* < 0.05 versus UUO+Vehicle group.

Subsequently, whether the supplementation of CTRP13 could influence renal inflammation in the UUO-model mice was investigated. As shown in Figure 6A, in the tubulointerstitial regions, the UUO-treated mice developed kidney inflammation with the infiltration of F4/80^+^ macrophages (Figure 6A). This was accompanied by a significant increase in the expression levels of MCP-1, IL-6, and IL-1β. However, ectopic CTRP13 infusion alleviated the UUO-exacerbated kidney inflammation, with the infiltration of fewer F4/80^+^ cells and the downregulation of MCP-1, IL-1β, and IL-6 (Figure 6B).

### 3.7. CTRP13 Suppresses NF-κB p65 Signaling Pathway in UUO-Model Mice

**Figure 7:**
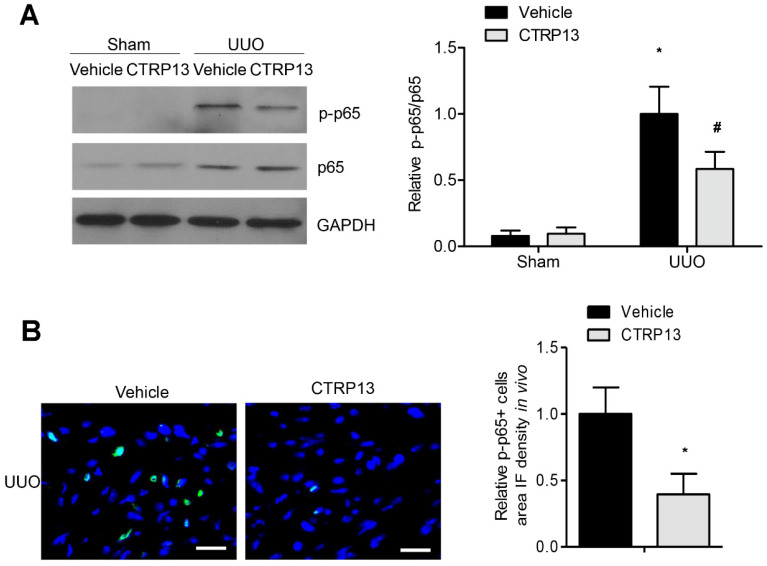
CTRP13 suppresses UUO-induced NF-κB p65 signaling pathway activation. (**A**) Representative Western blot analysis of P65 and phosphorylated p65 in the kidney of UUO mice. (**B**) Representative immunofluorescence staining of phosphorylated p65 in the kidney of UUO mice. Scale bar = 50 μm. * *p* < 0.05 versus Sham+Vehicle or Vehicle (UUO) group. # *p* < 0.05 versus UUO+Vehicle group.

The NF-κB signaling pathway has been reported to have a critical role in the progression of inflammation and renal fibrosis [19]. As shown in Figure 7A, the expression levels of p65 and phosphorylated p65 were markedly upregulated following UUO surgery. However, treatment with CTRP13 markedly disrupted the UUO-mediated upregulation of p65 phosphorylation. These results were corroborated by the results of the immunofluorescence experiments, which revealed that CTRP13 treatment mitigated the kidney inflammation that was associated with the suppression of NF-κB signaling, a finding that was also supported by a significant decrease in p65 phosphorylation in UUO-model mice (Figure 7B). Collectively, these data indicated that CTRP13 could mediate the inhibition of the UUO-induced NF-κB p65 signaling pathway, thereby inhibiting the recruitment of proinflammatory factors in the tubulointerstitial regions.

## 4. Discussion

In the present study, we first found that, in renal fibrosis, the serum level of CTRP13 decreased gradually with the development of kidney interstitial fibrosis. Ectopic CTRP13 infusion resulted in an inhibition of fibroblast activation, excessive deposition of ECM, and infiltration of proinflammatory cells, ultimately halting the development of UUO-induced renal interstitial fibrosis. The findings of the present study have provided new evidence for a vital role of CTRP13 in the progression of UUO-induced kidney inflammation and fibrosis. 

Regarding both acquired and congenital etiologies in patients with CKD, renal structural abnormalities and dysfunction are known to cause abnormally low levels of protein excretion and filtration [20]. Therefore, optimizing microcirculation, lowering the levels of urinary protein, and preventing the deterioration of kidney function are the critical objectives and means of the management of CKD [21]. Angiotensin receptor blockers, angiotensin-converting enzyme inhibitors, immunosuppressants and glucocorticoids are the agents primarily used to decrease urinary protein levels [22]. These medications are associated with different contraindications and side effects, although CTRP13 is currently gaining a lot of attention, as it has the advantage of exerting a comprehensive range of unique treatment effects, unlike the abovementioned pharmaceuticals. The findings of the present study have demonstrated that CTRP13 attenuates UUO-induced renal dysfunction via the TGF-β1/Smad and NF-κB p65 signaling pathways. It is anticipated that its protective effects may be generally applicable, working against fibrosis and inflammation due to the natural properties of CTRP13. Therefore, from a therapeutic and adverse-event standpoint, the present findings have potentially expanded the possibilities of administering CTRP13 therapy to multiple inflammatory and fibrosis diseases beyond obstructive nephropathy. 

CTRP13, an adipose-tissue-derived adipokine, has been reported to mediate glucose metabolism via activating the AMP-activated protein kinase (AMPK) signaling pathway [8]. In hepatocytes, CTRP13 has also been shown to increase lipid-induced insulin resistance through inhibiting stress-activated protein kinase/c-Jun N-terminal kinase stress signaling [8]. However, in CTRP13 knockout mice fed a high-fat diet, CTRP13 deficiency improved the systemic metabolic profiles as well as reduced adipose tissue inflammation and hepatic steatosis [23]. Recently, our group has shown that CTRP13 exerts multiple beneficial effects on cardiovascular disease [10,24,25,26]. In chronic coronary artery disease, ectopic CTRP13 infusion was shown to dramatically reduce the progression of atherosclerosis [25]. Furthermore, CTRP13 infusion was able to improve endothelial nitric oxide synthase (eNOS) coupling, thereby protecting the endothelial function in diabetic mice [24]. Interestingly, to the best of our knowledge, that study was the first to uncover a new and crucial role of CTRP13 in UUO-mediated renal dysfunction, as it demonstrated that CTRP13 has the effect of ameliorating tubulointerstitial fibrosis and tubular injury via the TGF-β1/Smad and NF-κB p65 signaling pathways. Moreover, in addition to CTRP13 and consistent with previous studies, the CTRP family members exert multiple effects on regulating the pathophysiological and physiological processes of the immune and metabolic systems [27,28]. In addition, the adipokine CTRP6 was shown to alleviate ischemia–reperfusion injury of the kidney by modulating the PI3K/Akt signaling pathway [29]. Furthermore, CTRP3 and CTRP6 have been reported to be potential therapeutic targets for diabetic nephropathy, exerting their effects via the regulation of glomerular mesangial cell function [30,31], indicating that the CTRP family of adipokines may be involved in the regulation of the urinary system. Future studies will need to be focused on whether other CTRP members can also participate in the progression of kidney inflammation and fibrosis. Developing therapies comprising a mixture of CTRPs may have potential therapeutic value in terms of overcoming the progression of chronic renal dysfunction. 

In the present study, it has been shown that CTRP13 can ameliorate UUO-induced kidney dysfunction via NF-κB-driven kidney inflammation and TGF-β1/Smad3-mediated renal fibrosis. Previous studies have reported that Smad7 is negatively regulated via Smad3 according to Arkadia- and Smurf2-associated ubiquitin degradation mechanisms [32,33]. Smad7 has been reported to be a suppressor of NF-κB signaling. The inactivation of NF-κB signaling mediated by Smad7 might serve as a critical ‘bridge’ that connects the protective effects on renal fibrosis and inflammation that result from treatment with CTRP13. Additionally, Smad3 has been shown to be capable of inducing the expression of MCP-1 via binding to the MCP-1 promoter [34]. In addition, inhibiting both MCP-1-dependent inflammatory cytokine production and the infiltration of macrophages might provide an alternative mechanism that underlies the observed protection of UUO-induced kidney inflammation mediated by CTRP13 in this study. In this work, we used recombinant CTRP13 protein infusion to produce protective effects on renal function. It is possible that CTRP13 may exert kidney protective effects by acting on other organs rather than the kidney directly. Next, constructing kidney-specific CTRP13 knockout mice will help us to test this hypothesis in the future.

In conclusion, in the present study, we have shown that CTRP13 fulfills an important role in UUO-mediated obstructive nephropathy. CTRP13 exerts its renal protective effects through inhibiting the TGF-β1/Smad and NF-κB signaling pathways. Therefore, these findings have provided a rationale for recruiting CTRP13 as a possible therapeutic approach for patients with renal inflammation and fibrosis.

## Data Availability

The data presented in this study are available on request from the corresponding author.

## Supplementary Materials

The following supporting information can be downloaded at https://www.mdpi.com/article/10.3390/biomedicines12010051/s1, Table S1: Plasma CTRP13 levels were decreased in the UUO mice with renal fibrosis; Table S2: Effects of CTRP13 treatment on plasma CTRP13, Scr, Bun, and 24 h UP levels in the UUO mice with renal fibrosis.

## References

[1] Yu H.T. (2003). Progression of chronic renal failure. Arch. Intern. Med..

[2] Tonelli M., Karumanchi S.A., Thadhani R. (2016). Epidemiology and Mechanisms of Uremia-Related Cardiovascular Disease. Circulation.

[3] Liu Y. (2006). Renal fibrosis: New insights into the pathogenesis and therapeutics. Kidney Int..

[4] Shabaka A., Casecs-Corona C., Fernandez-Juarez G. (2021). Therapeutic Insights in Chronic Kidney Disease Progression. Front. Med..

[5] Liu Y. (2011). Cellular and molecular mechanisms of renal fibrosis. Nat. Rev. Nephrol..

[6] Zheng Q., Yuan Y., Yi W., Lau W.B., Wang Y., Wang X., Sun Y., Lopez B.L., Christopher T.A., Peterson J.M. (2011). C1q/TNF-related proteins, a family of novel adipokines, induce vascular relaxation through the adiponectin receptor-1/AMPK/eNOS/nitric oxide signaling pathway. Arterioscler. Thromb. Vasc. Biol..

[7] Xie Y., Meng Z., Gao J., Liu C., Wang J., Guo R., Zhao J., Lopez B., Christopher T., Lee D. (2021). C1q Complement/Tumor Necrosis Factor-Associated Proteins in Cardiovascular Disease and COVID-19. Proteomes.

[8] Wei Z., Peterson J.M., Wong G.W. (2011). Metabolic regulation by C1q/TNF-related protein-13 (CTRP13): Activation OF AMP-activated protein kinase and suppression of fatty acid-induced JNK signaling. J. Biol. Chem..

[9] Bai B., Ban B., Liu Z., Zhang M.M., Tan B.K., Chen J. (2017). Circulating C1q complement/TNF-related protein (CTRP); CTRP9, CTRP12 and CTRP13 concentrations in Type 2 diabetes mellitus: In vivo regulation by glucose. PLoS ONE.

[10] Li Y., Wang W., Chao Y., Zhang F., Wang C. (2019). CTRP13 attenuates vascular calcification by regulating Runx2. FASEB J..

[11] Kim D., Lee A.S., Jung Y.J., Yang K.H., Lee S., Park S.K., Kim W., Kang K.P. (2014). Tamoxifen ameliorates renal tubulointerstitial fibrosis by modulation of estrogen receptor alpha-mediated transforming growth factor-beta1/Smad signaling pathway. Nephrol. Dial. Transplant..

[12] Choi D.E., Jeong J.Y., Lim B.J., Chang Y.K., Na K.R., Shin Y.T., Lee K.W. (2011). Aliskiren ameliorates renal inflammation and fibrosis induced by unilateral ureteral obstruction in mice. J. Urol..

[13] Wang J., Ge S., Wang Y., Liu Y., Qiu L., Li J., Huang X., Sun L. (2021). Puerarin Alleviates UUO-Induced Inflammation and Fibrosis by Regulating the NF-kappaB P65/STAT3 and TGFbeta1/Smads Signaling Pathways. Drug. Des. Devel. Ther..

[14] Mao Y., Zhang X., Peng W., Liu H., Zhou X., Liang L., Xiang J., Zhang H., Wang D., Liu L. (2021). EI24 alleviates renal interstitial fibrosis through inhibition of epithelial-mesenchymal transition and fibroblast activation. FASEB J..

[15] Wang C., Xu W., An J., Liang M., Li Y., Zhang F., Tong Q., Huang K. (2019). Poly(ADP-ribose) polymerase 1 accelerates vascular calcification by upregulating Runx2. Nat. Commun..

[16] Wang C., Zhang F., Wang L., Zhang Y., Li X., Huang K., Du M., Liu F., Huang S., Guan Y. (2013). Poly(ADP-ribose) polymerase 1 promotes oxidative-stress-induced liver cell death via suppressing farnesoid X receptor alpha. Mol. Cell Biol..

[17] Huang R., Fu P., Ma L. (2023). Kidney fibrosis: From mechanisms to therapeutic medicines. Signal Transduct. Target. Ther..

[18] Meran S., Steadman R. (2011). Fibroblasts and myofibroblasts in renal fibrosis. Int. J. Exp. Pathol..

[19] Zhang H., Sun S.C. (2015). NF-kappaB in inflammation and renal diseases. Cell Biosci..

[20] Chen T.K., Knicely D.H., Grams M.E. (2019). Chronic Kidney Disease Diagnosis and Management: A Review. JAMA.

[21] Wuhl E., Schaefer F. (2008). Therapeutic strategies to slow chronic kidney disease progression. Pediatr. Nephrol..

[22] Wilmer W.A., Rovin B.H., Hebert C.J., Rao S.V., Kumor K., Hebert L.A. (2003). Management of glomerular proteinuria: A commentary. J. Am. Soc. Nephrol..

[23] Chen F., Sarver D.C., Saqib M., Zhou M., Aja S., Seldin M.M., Wong G.W. (2023). CTRP13 ablation improves systemic glucose and lipid metabolism. Mol. Metab..

[24] Wang C., Chao Y., Xu W., Liang M., Deng S., Zhang D., Huang K. (2020). CTRP13 Preserves Endothelial Function by Targeting GTP Cyclohydrolase 1 in Diabetes. Diabetes.

[25] Wang C., Xu W., Liang M., Huang D., Huang K. (2019). CTRP13 inhibits atherosclerosis via autophagy-lysosome-dependent degradation of CD36. FASEB J..

[26] Xu W., Chao Y., Liang M., Huang K., Wang C. (2021). CTRP13 Mitigates Abdominal Aortic Aneurysm Formation via NAMPT1. Mol. Ther..

[27] Schaffler A., Buechler C. (2012). CTRP family: Linking immunity to metabolism. Trends Endocrinol. Metab..

[28] Seldin M.M., Tan S.Y., Wong G.W. (2014). Metabolic function of the CTRP family of hormones. Rev. Endocr. Metab. Disord..

[29] Xiang H., Xue W., Li Y., Zheng J., Ding C., Dou M., Wu X. (2020). C1q/TNF-related protein 6 (CTRP6) attenuates renal ischaemia-reperfusion injury through the activation of PI3K/Akt signalling pathway. Clin. Exp. Pharmacol. Physiol..

[30] Hu T.Y., Li L.M., Pan Y.Z. (2019). CTRP3 inhibits high glucose-induced human glomerular mesangial cell dysfunction. J. Cell Biochem..

[31] Xu E., Yin C., Yi X., Liu Y. (2020). Knockdown of CTRP6 inhibits high glucose-induced oxidative stress, inflammation and extracellular matrix accumulation in mesangial cells through regulating the Akt/NF-kappaB pathway. Clin. Exp. Pharmacol. Physiol..

[32] Koinuma D., Shinozaki M., Komuro A., Goto K., Saitoh M., Hanyu A., Ebina M., Nukiwa T., Miyazawa K., Imamura T. (2003). Arkadia amplifies TGF-beta superfamily signalling through degradation of Smad7. EMBO J..

[33] Kavsak P., Rasmussen R.K., Causing C.G., Bonni S., Zhu H., Thomsen G.H., Wrana J.L. (2000). Smad7 binds to Smurf2 to form an E3 ubiquitin ligase that targets the TGF beta receptor for degradation. Mol. Cell.

[34] Feinberg M.W., Shimizu K., Lebedeva M., Haspel R., Takayama K., Chen Z., Frederick J.P., Wang X.F., Simon D.I., Libby P. (2004). Essential role for Smad3 in regulating MCP-1 expression and vascular inflammation. Circ. Res..

