# Supplementary material for: Adiponectin C1q/Tumor Necrosis Factor-Related Protein 13 (CTRP13) Protects against Renal Inflammation and Fibrosis in Obstructive Nephropathy
Source: Biomedicines. 2023 Dec 25;12(1):51. doi: 10.3390/biomedicines12010051 (PMC10812933; doi:10.3390/biomedicines12010051)
Supplement: Supplementary file 1 [file biomedicines-12-00051-s001.zip › biomedicines-2729906-supplementary.pdf]

Supplementary Files

Table S1. Plasma CTRP13 levels were decreased in the UUO mice with renal fibrosis.

	Sham	3 days UUO	7 days UUO	14 days UUO
Serum CTRP13 concentration（ng/ml）	128.8±29.6	87.5±20.5*	64.8±13.9*	55.2±21.7*

Table S2. Effects of CTRP13 treatment on plasma CTRP13, Scr, Bun and 24-h UP levels in the UUO mice with renal fibrosis

	Sham+Vehicle	Sham+CTRP13	UUO+Vehicle	UUO+CTRP13
Plasma CTRP13 concentration （ng/ml）	139.6±21.6	180.2±13.1*	69.6±19.3*	146.2±15.6#
Plasma Scr concentration (mM/L)	35.8±7.7	37.2±15.6	80.3±13.2*	52.6±9.7#
Plasma BUN concentration (mM/L)	13.4±2.7	13.1±3.7	29.6±4.2*	19.4±4.8#
24-h UP (mg/day)	9.8±2.6	10.6±3.4	22.1±3.6*	14.2±2.4#

Values are presented as mean ± SD. Differences between groups were assessed by unpaired t-test or ANOVA with Bonferroni post hoc test. *p < 0.05 versus Sham or Sham+Vehicle group. #p < 0.05 versus UUO+Vehicle group. Abbreviations: BUN, blood urea nitrogen; 24 h UP, 24 hour urine protein; SCr, serum creatinine.
